# The Effect of Temperature Treatment on the Structure of Polyelectrolyte Multilayers

**DOI:** 10.3390/polym8040120

**Published:** 2016-04-02

**Authors:** Maximilian Zerball, André Laschewsky, Ralf Köhler, Regine von Klitzing

**Affiliations:** 1 Stranski-Laboratorium für Physikalische und Theoretische Chemie, Institut für Chemie, Technische Universität Berlin, Strasse des 17. Juni 124, 10623 Berlin, Germany; maximilian.zerball@mailbox.tu-berlin.de; 2 Fraunhofer Institute of Applied Polymer Research, Geiselbergstr. 69, 14476 Postdam-Golm, Germany; andre.laschewsky@iap.fraunhofer.de; 3 Institut für Chemie, Universität Postdam, Karl-Liebknecht Str. 24-25, 14476 Postdam-Golm, Germany; 4 Institut für weiche Materie und Funktionelle Materialien, Helmholtz-Zentrum Berlin, Hahn Meitner-Platz 1, 14109 Berlin, Germany; ralf.koehler@helmholtz-berlin.de

**Keywords:** polyelectrolyte multilayer, neutron reflectometry, internal structure, temperature, swelling behavior, odd–even effect, PSS/PDADMAC

## Abstract

The study addresses the effect of thermal treatment on the internal structure of polyelectrolyte multilayers (PEMs). In order to get insight into the internal structure of PEMs, Neutron Reflectometry (NR) was used. PEMs with a deuterated inner block towards the substrate and a non-deuterated outer block were prepared and measured in 1% RH and in D${}_{2}$O before and after a thermal treatment. Complementarily, PEMs with the same number of layers but completely non-deuterated were investigated by ellipsometry. The analysis for the overall thickness (*d*), the average scattering length density (SLD) and the refractive index (*n*) indicate a degradation of the PEM. The loss in material is independent of the number of layers, *i.e.*, only a constant part of the PEM is affected by degradation. The analysis of the internal structure revealed a more complex influence of thermal treatment on PEM structure. Only the outermost part of the PEM degenerates, while the inner part becomes denser during the thermal treatment. In addition, the swelling behavior of PEMs is influenced by the thermal treatment. The untreated PEM shows a well pronounced odd—even effect, *i.e.*, PDADMAC-terminated PEMs take up more water than PSS-terminated PEMs. After the thermal treatment, the odd-even effect becomes much weaker.

## 1. Introduction

Polyelectrolyte Multilayers (PEMs), which are nanoscale polymeric films prepared by the layer-by-layer technique, are interesting tools for applications and fundamental science [1,2,3]. The properties of PEMs can be affected by temperature treatment during [4,5,6,7,8] or after [9,10,11,12] the PEM preparation. In general, an increased temperature during PEM preparation increases the thickness increment per PE layer and extends the non-linear growing phase of non-linear growing PEMs [6,8]. In addition, linear growing PEM systems can be forced to a non-linear growth. The increase in temperature changes the balance between polyion-polyion and polyion-counterion interaction and increases the probability of polymer transport to the surface. The effect of thermal treatment after preparation is interesting for possible PEM containing devices, e.g., for devices designed for high operation temperatures. Furthermore, the understanding of the reaction of PEMs on thermal stress helps to better understand their nature.

The effect of temperature on PEMs was intensively investigated for microcapsules [9,10,13]. During the heating of PSS/PDADMAC microcapsules to temperatures over 65 ${}^{\circ }$C PSS-terminated capsules shrink, while PDADMAC-terminated capsules swell until they rupture. The different behavior of PSS-terminated and PDADMAC-terminated capsules was attributed to the different ratio of positive and negative charges inside the PEM capsules. The PDADMAC-terminated PEM capsules take up more water during heating to increase the distance between charges, while the more charge balanced PSS-terminated PEM capsules minimize the polymer water surface. Unfortunately, the behavior of PEM microcapsules cannot be transferred readily to PEMs attached on a solid substrate. Due to the fixation on the substrate, the PEMs are less flexible, provide a smaller surface and are sterically hindered. Microcapsules can respond with changes in wall thickness and changes in capsule diameter, while a PEM attached on a solid substrate can only react by changes in thickness. Furthermore, the influence of the substrate itself on the PEM is obvious but not understood yet. QCM-D measurements show an increasing swelling of PEMs with increasing temperature, but a rupturing was not reported [12]. For non-linear growing PSS/PDADMAC PEMs (prepared at ionic strength >0.1 mol/L) a glass transition temperature at about 50 ${}^{\circ }$C was determined. Linear growing PSS/PDADMAC PEMs (prepared without salt) did not show any transition up to 110 ${}^{\circ }$C [12]. Neutron reflectometry measurements after a thermal treatment revealed annealing effects related to a loss in swelling, which was detectable by a decrease in roughness and scattering length density (SLD) [11].

Temperature studies indicate a restructuring of the PEM [9,10,11,13,14,15,16]. The question arises whether the structure across the PEM perpendicular to the PEM surface changes during thermal treatment. While investigation of the average structure of PEMs is well established with a high variety of methods (ellipsometry, QCM-D, X-ray reflectometry, *etc.*), the access to internal properties is rather difficult. One powerful technique to probe internal properties is Neutron Reflectometry (NR) [17,18]. NR determines the scattering length density profile across the PEM. In order to get information about the internal structure of the film, one can change the contrast by controlled deuteration of the specific region, *i.e.*, a specific block [7,19,20,21,22]. Hence, different contrasts inside PEMs can be achieved without changing their chemical behavior. Thus, the effect of a thermal treatment on the internal structure can be traced by measuring the changes in thickness, SLD and roughness of the deuterated and non-deuterated block.

Complementary to neutron reflectometry, ellipsometry was used to investigate effects on thickness and refractive index before, after and during the temperature treatment. Non-linear growing PSS/PDADMAC PEMs of two different thicknesses are studied. In this context, thin refers to PEMs with 20 and 21 single layers while thick refers to PEMs with 36 and 37 single layers. In addition, the effect of thermal treatment is studied in dependence of polyion type (PSS or PDADMAC) of the outermost layer.

## 2. Materials and Methods

### 2.1. Chemicals

Polyethyleneimine (PEI, ${\bar{M}}_{n}$≈ 60 kDa determined by GPC ${\bar{M}}_{w}$≈ 750 kDa determined by LS) and Polystyrenesodiumsulfonate (PSS, ${\bar{M}}_{w}$ = 70 kDa), were purchased from Sigma-Aldrich (Steinheim, Germany) and were used without further purification. Fully deuterated PSS (dPSS ${\bar{M}}_{w}$ = 78.3 kDa Đ< 1.2 by GPC) was purchased from Polymer Standard Service GmbH (Mainz, Germany). Poly(diallyldimethylammoniumchloride) (PDADMAC, ${\bar{M}}_{w}$ = 135 kDa Đ= 1.8 determined by GPC and ${}^{1}$H NMR) was synthesized by free radical polymerization of diallyl-dimethyl-ammonium chloride in aqueous solution, as described in a previous work [23]. The silicon wafers were a gift from Wacker Chemie AG (München , Germany). The silicon blocks were purchased by Silizium Bearbeitung A. Holm (Tann, Germany).

### 2.2. Layer-by-Layer Deposition

For the ellipsometric measurements, the multilayers were built on silicon wafers via the layer-by-layer method introduced by Decher [19]. All preparation steps were done by an automatic dipping device (Riegler & Kirstein, Berlin, Germany). The silicon wafers were cleaned for 30 min in a 1:1 mixture of 98% H${}_{2}$SO${}_{4}$/ 35% H${}_{2}$O${}_{2}$ and then rinsed with Milli-Q water . Afterwards, the wafers were covered by a precursor layer of PEI. PEI was deposited to the surface by immersing the wafers for 30 min into an aqueous solution containing $10^{-2}$ monomol/L (concentration based on monomer unit) PEI. Because of PEI, the PEMs become more compact and less rough [24]. Then, the wafer is alternately immersed into aqueous (Milli-Q water) polyelectrolyte solutions containing $10^{-2}$ monomol/L of the respective polyelectrolyte and 0.1 mol/L NaCl. Every polyelectrolyte layer was adsorbed for 20 min. After every adsorbed layer, the samples were rinsed three times for 1 min in Milli-Q water. After completion of the multilayer assembly, the wafers were dried in air. The polyelectrolyte solutions contained $10^{-2}$ monomol/L of the respective polyelectrolyte and 0.1 mol/L of NaCl. The preparation started with a PSS layer followed by a PDADMAC layer. Four different PEMs were prepared: two *thin* PEMs with 20 layers ((PSS/PDADMAC)${}_{10}$) or 21 layers ((PSS/PDADMAC)${}_{10}$/PSS), and two *thick* PEMs with 36 layers ((PSS/PDADMAC)${}_{18}$) or 37 layers ((PSS/PDADMAC)${}_{18}$/PSS). For the neutron reflectivity measurements, the samples were prepared on a silicon block ( 80 × 50 × 15 mm${}^{3}$) substituting PSS in the first 6 bilayers by dPSS. Otherwise, the preparation was the same as for the ellipsometric samples. In the following, the neutron reflectivity samples are named by the number of deuterated bilayer ($D_{x}$) and protonated bilayer ($H_{y}$), while half numbers indicate an additional layer of PSS on top. For example, the sample PEI/ (dPSS/PDADMAC)${}_{6}$/ (PSS/PDADMAC)${}_{4}$/ PSS is named as D6H4.5.

The layer numbers of thin and thick PEMs were chosen in consideration of the limits of internal structure and of the NR measurements, respectively. The preparation of thin PEMs is limited by the creation of a complete block structure. Polyelectrolytes within PEMs strongly interdigitate. Too thin deuterated blocks are completely mixed with non-deuterated material. Soltwedel et al. showed that the inner block should have at least 5 double layers and the outermost block at least 3 double layers [22]. To be sure to prepare a block structure PEMs with 6 deuterated bilayer and 4 non-deuterated bilayers were prepared. The preparation of thick PEMs is limited by the specifications of the neutron reflectometer. The V6 can resolve depth profiles of about 200 nm depth. Therefore, the thickest samples were prepared 10%–20 % thinner than 200 nm.

### 2.3. Ellipsometry

The thickness (*d*) and refractive index (*n*) of PEMs are determined by measuring the change of polarization of a laser beam reflected at the surface of the sample [25]. Ellipsometric measurements were performed with a PCSA (polarizer-compensator-sample-analyzer) ellipsometer (Optrel GbR, Sinzing, Germany). The experiments were carried out at a constant wavelength of 632.8 nm. For measurements against air the angle of incidence was fixed to $70^{\circ }$ (near the Brewster angle of the Si/air interface), while for measurements in water the angle of incidence was fixed to $60^{\circ }$ (near the Brewster angle of the Si/water interface). The change of polarization is defined by the ellipsometric angles Δ and Ψ, whereby Δ represents the change in phase shift and Ψ the change in ratio of amplitudes between the s-polarized and p-polarized component due to reflection. Δ and Ψ are converted into thickness and refractive index by the software “Ellipsometry: simulation and data evaluation” (Optrel, v. 3.1). A one-box model for the PEMs were assumed, the continuum media were air (*n* = 1.000) and silicon (*n* = 3.885; *k* = −0.180). The thin SiO${}_{x}$ layer on top of the silicon support was fixed with *n* = 1.459 [26], *d* = 1.5 nm. As fitting parameters the thickness and the refractive index of the PEMs were chosen. The average thickness *d* = 1.5 nm of the SiO${}_{x}$ layer was determined by measuring five individual Si Wafers with ellipsometry and X-ray reflectometry after the etching in H${}_{2}$O${}_{2}$/H${}_{2}$SO${}_{4}$.

The following measuring procedure was carried out. Initially, the sample was measured in dry N${}_{2}$ (1% RH) followed by a measurement in water. Then, the sample was kept for 2 h at 65 ${}^{\circ }$C. During the temperature treatment, Δ and Ψ were monitored. After the temperature treatment, the PEM was first measured in water and then in dry N${}_{2}$ (1% RH). To measure at 1% RH, a special measurements cell was constructed. The cell was flushed by a constant flow of dry N${}_{2}$ (1% RH). The samples were equilibrated for 20 min at 1% RH. The relative humidity was measured and recorded by a Testo 6681 Humidity measuring transducer with testo 6614 sensor (Testo Ag, Lenzkirch, Germany). For ellipsometry measurements in water, the ellipsometer was equipped with two light guides to avoid errors because of refraction at the air/water interface [27]. To be sure that the sample was fully equilibrated in water, Δ and Ψ were observed at one position of the sample until a constant value of the ellipsometric angles was reached. Then, the samples were measured at five different positions. During the temperature treatment, the ellipsometric angles were monitored over time at one spot. For the evaluation of the data, the above described model was used but with water (*n* = 1.332) instead of air as continuum. The decrease of $n_{H_{2}O}$ during the temperature treatment is smaller than 0.3% and can be neglected. The variation of PEM thickness and refractive index is determined by measuring 5 different spots on the sample. This is done for all applied conditions, *i.e.*, in the dried and swollen states, before and after the thermal treatment.

### 2.4. Neutron Reflectometry

The specular reflectivity (*R*) of neutrons on a surface covered with a PEM is measured in dependence of the momentum transfer ($Q_{z}$). $Q_{z}$ is related to the wavelength (λ = 4.66 Å) and angle of incident $\alpha_{i}$ by:

(1) $$Q_{z}=4\pi /\lambda sin\left(\alpha_{i}\right)$$

For the neutron reflectometry measurements, $Q_{z}$ was varied between 0.004 and 0.015 Å${}^{-1}$. The measurements were carried out at the V6 reflectometer at Helmholtz Zentrum Berlin [28]. Measurements at 1% RH were performed in a self-made humidity cell, the humidity was adjusted by a smooth stream of dried nitrogen and monitored by a humidity sensor (Hygropalm-HP22-A with HC2-P05 Sensor, Rotronic AG, Bassersdorf, Switzerland). The measurements in D${}_{2}$O were performed in a home-made liquid cell. The samples were equilibrated for at least 0.5 h. The temperature treatment was executed by heating the liquid cell for 2 h at 65 ${}^{\circ }$C. The NR samples were treated analogous to the ellipsometric samples. First, the sample was measured in dried N${}_{2}$. Afterwards, the sample was placed in the liquid cell and measured in D${}_{2}$O. After the measurement in D${}_{2}$O, the sample was kept for 2 h at 65 ${}^{\circ }$C. After the temperature treatment the sample was measured in D${}_{2}$O and then in dried air.

From the measured reflectivity curves, the SLD profile across the PEM can be extracted by using a least mean-squares fitting routine. The data were fitted using the Motofit Package for Igor Pro [29]. The algorithm splits the measured PEM into boxes of constant SLD and thickness with a Gaussian roughness between each box. To prevent artifacts in the SLD profile, it is important that the roughness of one box does not exceed 1/3 of the box thickness. This is not an issue for the interface between the outermost non-deuterated block and the environment but for the interface between deuterated block and non-deuterated block. Therefore, the deuterated part and the transition region between deuterated and non-deuterated block were split into a varied number of boxes with a fixed thickness of 2 nm and a roughness of zero between the boxes. As an additional constraint, the SLD decreases monotonically towards the outer interface of the PEM. The continuum media were silicon (SLD = 2.07 × 10${}^{-6}$ Å${}^{-2}$) and air (SLD = 0.00 × 10${}^{-6}$ Å${}^{-2}$) or D${}_{2}$O (SLD = 6.36 × 10${}^{-6}$ Å${}^{-2}$); the silicon oxide layer was fixed at SLD = 3.47 × 10${}^{-6}$ Å${}^{-2}$ , *d* = 1.5 ± 0.2 nm; σ = 0.3 ± 0.1 nm. From the SLD profiles, the overall thickness was extracted by summarizing the thickness of all boxes. The average SLD was calculated by the area under the SLD profiles divided through the overall thickness.

## 3. Results

### 3.1. The Effect of Thermal Treatment on the Average Structure of PEMs

Table 1 shows the results of ellipsometric and neutron reflectometric measurements. The PEMs were measured in dried nitrogen (1% RH) and in water (H${}_{2}$O for ellipsometry and D${}_{2}$O for NR), before (b.t.) and after (a.t.) the thermal treatment. The neutron reflectivity curves are discussed in detail in the next section. Although ellipsometry and neutron reflectometry were carried out with different samples, the measured thicknesses are in good agreement within the expected error. At 1% RH, the thick PEMs (36/37 layers) are thicker than the thin PEMs (20/21 layers). Furthermore, the PSS-terminated samples are slightly thicker than the PDADMAC-terminated ones because of the additional layer. The refractive index of all samples is the same, which indicates a similar density of all samples. The average SLD of the PSS-terminated samples is slightly higher than the average SLD of the PDADMAC-terminated samples; this is because of a slightly higher PSS/PDADMAC ratio of the PSS-terminated samples and the fact that the SLD of PSS (SLD ≈ 2 × 10${}^{-6}$ Å${}^{-2}$) is higher than the SLD of PDADMAC (SLD ≈ 0.5 × 10${}^{-6}$ Å${}^{-2}$). The average SLD of the thin PEMs is higher than the one of the thick PEMs because of the relative larger amount of deuterated layers.

After the thermal treatment, thickness, refractive index and SLD of all samples decrease in 1% RH. A decrease in SLD and refractive index is typical for a decreased density. If accompanied with a decreased thickness, a decreased density indicates a loss of material. The absolute decrease in thickness caused by the thermal treatment is the same for all samples, *i.e.*, irrespective of the type of outermost layer and the number of layers. According to the NR data, the absolute loss in thickness is about 9 nm for all samples. The ellipsometric data also show constant though lower decreased thickness of about 3 nm after the thermal treatment. Obviously, the thermal treatment affects only a constant outermost part of the PEM.

Complementary to the measurements at 1% RH, the PEMs were investigated in water (H${}_{2}$O for ellipsometry and D${}_{2}$O for NR) before and after the thermal treatment. Before the thermal treatment, the PEMs swell as earlier reported [30,31]. The thickness of the PDADMAC-terminated PEMs increases more as the thickness of the PSS-terminated PEMs, the refractive index behaves oppositely, *i.e.*, the PDADMAC-terminated PEMs takes up more water than the PSS-terminated PEMs. This phenomenon is called odd–even effect, and it is intensively discussed in literature [30,31,32,33,34]. The values for thickness and refractive index in dried and swollen state are in good agreement with recently published results [31].

The discussion about the swollen PEMs after the thermal treatment has to be distinguished between thin (20/21 layers) and thick (36/37 layers) PEMs. The swollen thin PEMs show after the thermal treatment a slightly lower thickness than before the thermal treatment. The average SLD of the thin samples measured in D${}_{2}$O is higher after the thermal treatment than before, while the refractive index behaves the opposite. Both indicate a higher water uptake and consequently lower density of the PEM. The lower density together with the slightly lower thickness indicate a loss of material, which is in agreement with the results from the measurement in 1% RH.

The swollen thick PEMs exhibit a lower thickness after thermal treatment. In opposition to the thin samples, the average SLD of the swollen thick PEMs decreases after the thermal treatment, while the refractive index increases. The decreased thickness together with the decrease of the average SLD, and the increase of the refractive index indicate an increased density. The change in average SLD and refractive index is much more pronounced for the thick PDADMAC-terminated sample. In contrast, dried PEMs behave oppositely, the change in thickness, SLD and refractive index indicate decreases in density during the thermal treatment.

For a better understanding of the swelling process, a more detailed examination of the swelling behavior before and after the thermal treatment is necessary. The total amount of water, which is absorbed by a PEM during the swelling process is separated into swelling water and void water. Swelling water increases the thickness of the PEM, void water does not influence the thickness but the SLD of the PEM. The neutron data are used to calculate the swelling water and void water of the PEMs in the following manner:

The swelling water is calculated by:

(2) $$\phi_{\mathrm{swell}}=\frac{d_{\mathrm{swollen}}-d_{\mathrm{dry}}}{d_{\mathrm{swollen}}}$$

where $d_{\mathrm{swollen}}$ is the thickness in D${}_{2}$O and $d_{\mathrm{dry}}$ the thickness of the dried PEM. According to previous publications [31,35,36,37], the amount of void water depends on the polymer fraction *x* of the dried PEM, which is calculated in the following manner:

(3) $$x=\frac{SLD_{\mathrm{dry}}}{SLD_{D_{2}O}}-\frac{SLD_{\mathrm{swollen}}-\phi_{\mathrm{swell}}SLD_{D_{2}O}}{\left(1-\phi_{\mathrm{swell}}\right)\left(SLD_{D_{2}O}\right)}+1$$

where $SLD_{\mathrm{dry}}$ is the SLD of the dried PEM, $SLD_{\mathrm{swollen}}$ is the SLD of the swollen PEM and $SLD_{D_{2}O}$ the SLD of D${}_{2}$O (6.36 × 10${}^{-6}$ Å${}^{-2}$). The model assumes that the dry PEM has a volume ratio of voids (1 −*x*) which is filled with vacuum. Due to the fact that the PEM swells by Φ${}_{\mathrm{swell}}$, the volume ratio of the voids Φ${}_{\mathrm{void}}$ decreases by factor (1 − Φ${}_{\mathrm{swell}}$). Hence, the amount of void water inside the swollen PEM is calculated by:

(4) $$\phi_{\mathrm{void}}=\left(1-\phi_{\mathrm{swell}}\right)\left(1-x\right)$$

The role of void water inside PEMs was recently reviewed [37]. Furthermore, a method to calculate the amount of void water from ellipsometry data was recently reported [31]. Because of the low number of individual samples and the lower accuracy of void water calculated from ellipsometry data, void water and swelling water are only calculated from NR data in this study.

Table 2 shows the swelling water $\phi_{\mathrm{swell}}$ and void water $\phi_{\mathrm{void}}$ of the investigated PEMs calculated from NR data. As already mentioned, the water content in PDADMAC-terminated PEMs is higher as in PSS-terminated PEMs. The amount of void water before the thermal treatment agrees with previous studies [31,35]. After the thermal treatment the water content of the thin samples increases strongly in comparison to the water content before the thermal treatment. Furthermore, the amount of void water decreases, but the difference is within the error margin. The strong increase in absorbed water indicates a lower density of the PEMs. Furthermore, the difference in swelling water between the thin PDADMAC-terminated PEM and the PSS-terminated PEM decreases from 9% before the thermal treatment to 5% after the thermal treatment, which indicates a weaker odd-even effect between the thin PDADMAC-terminated and PSS-terminated PEMs. The effect of the thermal treatment on the amount of swelling water inside the thick PEMs is less pronounced than for the thin PEMs. The water content of the thick PSS-terminated PEM is constant within the error margin, while the water content of the thick PDADMAC-terminated PEM decreases after the thermal treatment. The amount of void water decreases for both the PSS-terminated and the PDADMAC-terminated PEM. In addition, the differences in swelling water between the thick PDADMAC-terminated PEM and the PSS-terminated PEM decreases to zero, which indicates a vanishing of the odd-even effect between the thick PDADMAC-terminated and PSS-terminated PEMs. The effect of the thermal treatment on the odd-even effect is discussed later in detail.

**Figure 1:**
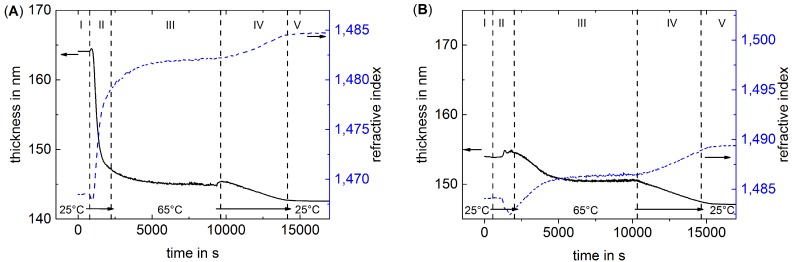
Change of thickness and refractive index over time during the temperature treatment measured in water by ellipsometry of samples with 36 layers (**A**) and 37 layers (**B**). The measurement begins at 25 ${}^{\circ }$C, then the temperature is increased to 65 ${}^{\circ }$C where it is maintained for 2 h. Then, the temperature was decreased back to 25 ${}^{\circ }$C

Due to the speed of a single ellipsometry measurement, it is possible to carry out kinetic measurements of PEMs during the temperature treatment. Figure 1 shows exemplary the change of thickness and refractive index over time during the temperature treatment of the two thick samples swollen in water. The measurements are divided into five phases: (I) starting phase at 25 ${}^{\circ }$C; (II) heating phase with increasing temperature up to 65 ${}^{\circ }$C; (III) constant temperature phase for 2 h at 65 ${}^{\circ }$C; (IV) cooling phase with decreasing temperature down to 25 ${}^{\circ }$C; (V) final phase with constant 25 ${}^{\circ }$C.

For each constant temperature regime (phase I, III, V), a plateau value for both thickness and refractive index is reached. From 25 to 65 ${}^{\circ }$C, the refractive index increases and the thickness decreases. After cooling down to 25 ${}^{\circ }$C, the thickness further decreases and the refractive index increases. The described changes in thickness and refractive index due to thermal treatment are independent of the outermost layer. Nevertheless, the change is more pronounced for the PDADMAC-terminated PEM. Therefore, the difference in swelling water between PSS-terminated and PDADMAC-terminated PEMs is after the temperature treatment less than before. The changes in thickness and refractive index are monotonous during cooling, while they are non-monotonous during heating. The over/undershoot of thickness and refractive index indicate a short term swelling of the PEM after starting to heat, which is more pronounced for the 37 layers thick PEM.

In summary, the measurements of the average structure of the PEMs show that both thickness and refractive index of dried samples is decreased after thermal treatment, what indicates degradation. Obviously, the degradation affects only a constant outermost part of the PEM. Therefore, the thin PEMs are more strongly affected from the relative loss of thickness by the thermal treatment, which is detectable by a stronger relative decrease in thickness, refractive index and SLD. Furthermore, the amount of swelling water strongly increases the thermal treatment. The thick samples are relatively less affected by the degradation. Furthermore, the thick samples show hints of a PEM densification. In the swollen state, the refractive index increases and SLD decreases. An additional hint for densification of the samples is given by the decreased amount of void water after the thermal treatment. Obviously, both loss in material and densification takes place during thermal treatment, and the whole process of thermal treatment is more complex as previously studies described [10,11]. It is assumed that densification and degradation take place in different regions of the PEM.

### 3.2. The Effect of Thermal Treatment on the Internal Structure of PEMs

**Figure 2:**
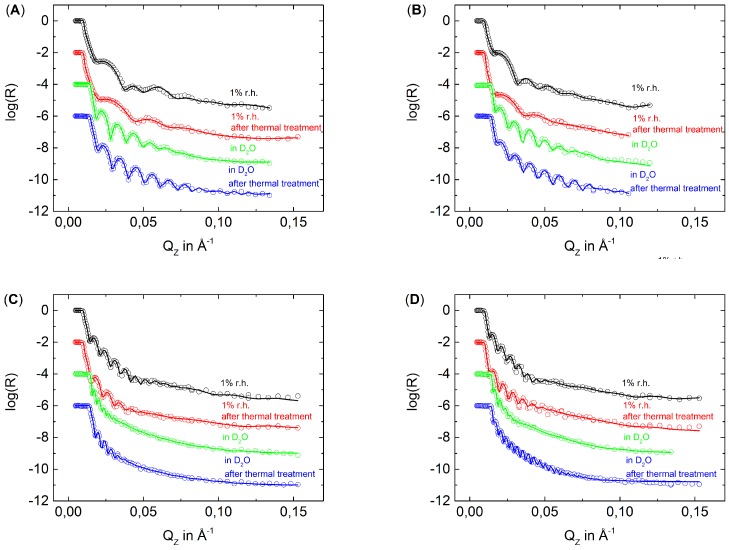
Neutron data of samples D6H4 (**A**); D6H4.5 (**B**); D6H12 (**C**) and D6H12.5 (**D**). The circles represent the measured data while the solid lines correspond to data fits. For the sake of clarity, the data were shifted by a value of −2(log(0.01)), −4 and −6.

**Figure 3:**
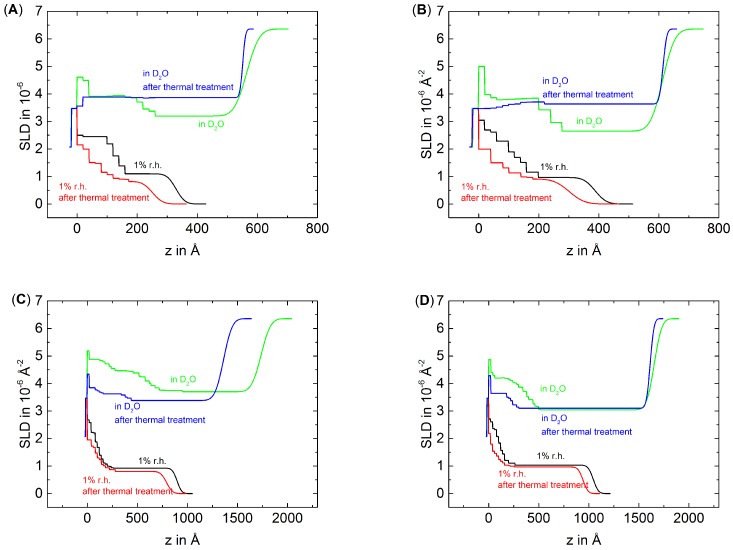
SLD profiles of D6H4 (**A**); D6H4.5 (**B**); D6H12 (**C**) and D6H12.5 (**D**) according to the fitted reflectivity data shown in Figure 2, whereby the SiO${}_{x}$/PEM interface is defined as *z* = 0.

Figure 2 shows the NR data fitted by an n-box model (between 20 and 40 boxes) of the thin samples D6H4, D6H4.5 and of the thick samples D6H12 and D6H12.5 measured at 1% RH and in D${}_{2}$O before and after thermal treatment. Figure 3 shows the resulting SLD profiles of the measured samples. For better comparability to the ellipsometric data, the SiO${}_{x}$/PEM interface is defined as *z* = 0. The measurements carried out in 1% RH show the expected SLD profile, with a higher SLD of the inner block and a lower SLD of the outermost block. Furthermore, the PEMs show nearly the same roughness in dried nitrogen, *i.e.*, there is no roughness odd-even effect as recently observed [31]. After the thermal treatment, the samples D6H12 and D6H12.5 show in 1% RH nearly the same roughness as before the treatment. In contrast, the samples D6H4 and D6H4.5 show after the thermal treatment a higher roughness than before the thermal treatment. The samples D6H4 and D6H4.5 are also the more degraded samples. Apparently, the strong degradation increases the roughness of dried PEMs. The measurements in D${}_{2}$O show that before the thermal treatment, the roughness of the PEMs increases due to the swelling. More water is absorbed by the PDADMAC-terminated PEMs than by the PSS-terminated PEMs. Consequently, the roughness of PDADMAC-terminated PEMs also increases more strongly during swelling. After the thermal treatment, the roughness measured in D${}_{2}$O decreases for all samples *i.e.*, the thermal treatment causes a smoothening of the surface. A smoothening of the surface is an indication for a densification of the PEMs.

**Figure 4:**
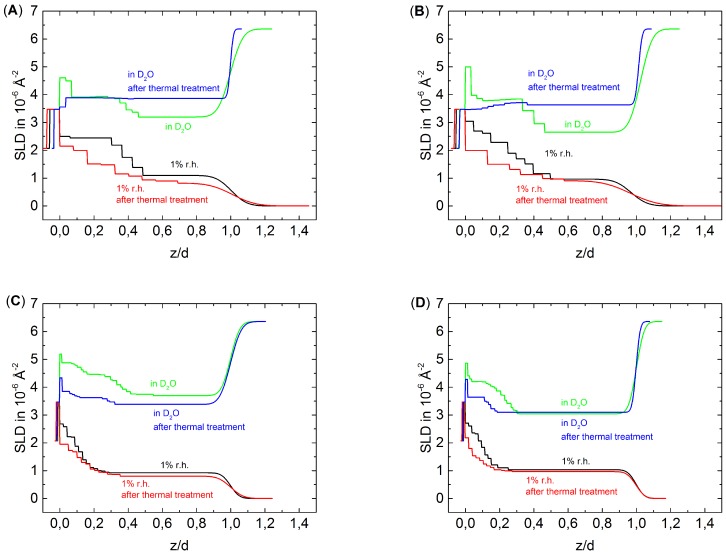
SLD profiles normalized to the total thickness *d* of D6H4 (**A**);D6H4.5 (**B**); D6H12 (**C**) and D6H12.5 (**D**).

For easier comparison of the different blocks, the distance *z* from substrate was normalized to the total thickness of the respective PEM *d* (Figure 4), which corresponds to the total PEM thickness shown in Table 1. After the thermal treatment of all PEMs, the block structure is still observable in 1% RH, although less pronounced than before the thermal treatment. The effect is more pronounced for the thin PEMs D6H4 and D6H4.5 than for the thick PEMs D6H12 and D6H12.5. Apparently, the deuterated part of the PEM mixes with the non-deuterated part of the PEM at least partially. Furthermore, the average SLD decreases, which suggests a loss of material.

The discussion of the effect of thermal treatment on swollen PEMs has to distinguish between thin and thick PEMs. Surprisingly, the block structure of thin PEMs is not observable after the thermal treatment. In comparison to the measurement in D${}_{2}$O before the thermal treatment, the average SLD of the inner block decreases slightly while the SLD of the outer block increases. In respect to the dried PEMs, the increase in SLD due to swelling increases of the outermost block but decreases of the innermost block after the thermal treatment, *i.e.*, the inner block swells less than the outermost block. Consequently, it is assumed that the temperature affects the inner and outermost part differently, whereas the term ”part” is used in a general case and does not necessarily match our block size. The treatment increases the density of the inner part, while the density of outer part decreases due to degradation.

The swollen thick PEMs preserve the block structure after the thermal treatment but much less pronounced than before the thermal treatment. The outermost block of the PSS-terminated PEM shows after the thermal treatment a slightly higher SLD than before the thermal treatment, while the SLD of the inner block decreases after the thermal treatment. The outermost block of the PDADMAC-terminated PEM shows, after the thermal treatment, a slightly lower SLD than before the thermal treatment, while the SLD of the innermost block decreases strongly due to the thermal treatment. In both cases, the SLD difference between the inner and outer block decreases in comparison to the measurement in D${}_{2}$O of the non-treated PEMs. This is also because of densification of the inner part and degradation of the outermost part of the PEMs.

## 4. Discussion

### 4.1. Degradation and Densification of the PEMs

The analysis of the average PEM structure indicates that two processes affect the PEMs during temperature treatment, degradation and densification. The analysis of the internal PEM structure indicates a partial degradation of the outermost part of the PEM and densification of the inner part.

The degradation of the PEM is indicated by the fact that the average SLD, refractive index and thickness of the PEMs measured in dried state decrease after the thermal treatment. Furthermore, it was concluded that the degradation affects only the outermost part of the PEM, which is detectable by two observations. First, the absolute loss in thickness is about 9 nm for all samples independent of the number of layers and the type of the outermost layer. Furthermore, the refractive index decreases at the same time, which is a clear hint for material loss. Kinetic ellipsometry measurements show that the thickness and refractive index of the PEMs reach a constant value during the constant temperature regime of 65 ${}^{\circ }$C, *i.e.*, the equilibrium is reached. Second, the neutron data show that mainly the outermost block of the partially deuterated PEMs is affected by degradation. The analysis of the SLD shows that thermal treatment causes a stronger swelling of the outer block than of the inner block. To summarize, these results indicate a degradation of the outer zone of all PEM.

The densification is indicated by a decrease of SLD and an increase of refractive index of the thick swollen PEMs after the thermal treatment, which indicates a lower amount of absorbed water and consequently a higher density. It is concluded for all samples that the densification affects mainly the inner part of the PEMs, which is detectable by a stronger swelling of the outer block in comparison to the inner block after the thermal treatment. In addition, the partial mixing of the deuterated and non-deuterated block provides an indirect indication for densification. Densification presupposes an increased mobility of PE chains so that the PE chains can arrange into a more compact conformation. In addition, earlier studies reported densification of PEMs [11]. Steitz *et al.* heated PSS/PDADMAC PEMs up to 40 ${}^{\circ }$C. The PEMs showed lower SLD in D${}_{2}$O and a decreased roughness but no degradation of the PEM took place. Apparently, degradation of the PEM only appears above a critical temperature, while densification always takes place. In the present study, the PEMs were heated up to 65 ${}^{\circ }$C, which is obviously high enough to cause degradation. An effect of the temperature on the amount of absorbed water was also detected for PLL/case in PEMs [38].

The degradation of a constant outermost part suggests a correlation with the type of growth. Most of the PEMs initially grow non-linear until a transition point is reached where the growth changes to a linear one. In addition, the investigated PEMs show an initial non-linear growth. The non-linear to linear growth transition is at about 20 layers [30,31]. Consequently, the thin samples are close to the transition point while the thick samples are clearly in the linear growing regime. The currently most favored approach to describe the non-linear to linear growth transition is to assume that the PEM as divided into an outermost less compact diffusion zone and a denser buried restructured zone [39]. The diffusion zone initially grows non-linearly until a critical thickness is reached, where the diffusion zone does not grow anymore. If the diffusion zone reaches its maximal thickness, the deepest buried layers begin to form the restructured zone, which grows linear. Apparently, the thermal treatment affects mainly the diffusion zone. The outer part of the diffusion zone is degenerated by the thermal treatment, while the inner part restructures and increases the size of the restructured zone. In a recent review, Volodkin *et al.* suggest that the charges in the diffusion zone are mainly extrinsically compensated, while the charges in the restricted zone are intrinsically compensated [40]. In the restructured zone, the polyelectrolyte chains are maximal crosslinked because of direct polyion-polyion interaction. In the diffusion zone, the amount of polyions-polyion bindings is lower. Instead, the charges are extrinsically compensated due to polyion-counterion interaction. Therefore, in this region, the PEM is softer, and less stable. An increase in temperature changes the balance between polyion-polyion interaction and polyion-counterion interaction towards the polyion-counterion interaction. Therefore, in the diffusion zone the number of polyion-polyion connections decreases and a dissolving of polyelectrolytes become more likely. Ghostine *et al.* showed that PSS/PDADMAC PEMs contain extrinsic binding sites [30]. The amount of negative binding sites is constant and independent of the outermost layer after 16 single layers (for PEMs prepared with 0.1 mol/L NaCl), while the amount of positive extrinsic binding sites depends on the layer number and the chemical nature of the outermost layer. The amount of positive extrinsic binding sites increases in a zig-zag shape, PDADMAC-terminated PEMs contain more positive extrinsic binding sites, than PSS-terminated PEMs. The latter shows only positive extrinsic binding sites after about 20 layers. Because the degradation appears in a constant part of the PEM, it is obvious that, for the degradation process, only the existence of extrinsic charge compensation is essential, and the amount of extrinsic binding sites has no effect on the total amount of lost material.

The block structure of the PEMs after the thermal treatment is less pronounced than before the thermal treatment but still present, *i.e.*, either only a part of the material inside the PEM is capable to freely move through the polymer matrix, or the restructuring process is not finished after 2 h (in opposite to the degradation process). The kinetic ellipsometry measurements show that the densification process is finished within the time of the temperature treatment. The refractive index and thickness show a constant value at the end of the constant temperature regime at 65 ${}^{\circ }$C. Therefore, the assumption of a higher mobility of only a part of the PEM seems more appropriate. Furthermore, this assumption is in agreement with the theory that the restructured zone grows due to the thermal treatment. The incorporation of non-deuterated material into the deuterated block would decrease the average SLD even if the material becomes denser. In summary, the restructured zone grows due to thermal treatment, while the diffusion zone degenerates.

### 4.2. The Weakened Odd-Even Effect

PSS/PDADMAC PEMs show an odd-even effect. PDADMAC-terminated PEMs take up more water than PSS-terminated PEMs. The odd-even effect is caused by the higher amount of positive extrinsic binding sites inside PDADMAC-terminated PEMs [30]. Surprisingly, the odd-even effect appears only if the PEM is immersed into liquid water, but not at high RH [31]. The reason for this difference could be that, only in water, the ions in the polyion-counterion binding dissociate, thus inducing an osmotic pressure. The osmotic pressure is compensated by a higher water uptake.

The odd-even effect can also be created during the PEM build-up, especially if at least one of the polyelectrolytes is a weak one [41,42,43], for short chain polyelectrolytes [44], at high salt concentration or if large ions are added [45]. In these studies, it could be shown that the complexation between the adsorbing polyelectrolyte and the formerly adsorbed polyelectrolyte is stronger than their adsorption to the PEM surface. In the present study, both polyanions and polycations are strong, and the PEMs are prepared at intermediate salt concentrations (0.1 mol/L NaCl). This system does not show any degradation in presence of one of the polyelectrolyte solutions [44]. The odd-even effect cannot be explained by long-range interactions with the substrate due to two reasons: (1) Branched PEI was used as the first layer, which overcompensates for the charge immediately and levels off all substrate effects as potential and roughness *etc.*; (2) Even in the case that the surface potential of the substrate would be partially present, the interaction is strongly screened due to the low dielectric permittivity ($\epsilon_{r}$ about 20–30) of the PEM [46,47] and the presence of counter ions. The Debye length is quite short (in the range of nanometers). In the present paper, the odd-even effect is a water swelling effect.

The results indicate a weakening of the odd-even effect due to the thermal treatment. For the thick samples, the weakening of the odd-even effect is obvious. The amount of absorbed water of the PDADMAC-terminated PEM decreases strongly after the thermal treatment, while the amount of absorbed water of the PSS-terminated PEM does not change. Before the thermal treatment, the PDADMAC-terminated PEM absorbs 10% more water than the PSS-terminated PEM. After the thermal treatment, the difference is close to zero. For the thin PEMs, the weakening of the odd-even effect is less obvious because the amount of swelling water increases for both PEMs. Nevertheless, the difference decreases. Before the thermal treatment the difference in water uptake between the PDADMAC-terminated and the PSS-terminated PEM is 9%, after the thermal treatment the difference is only 5%, *i.e.*, the odd-even effect becomes weaker. Furthermore, the relative degradation of the 20 layers thick PEM is stronger than the relative degradation of the 21 layers thick sample (25% for 20 layers 21% for 21 layers). Because the degradation also causes a lower density of the PEM, a more degraded PEM also absorbs more water, *i.e.*, the reduced water uptake caused by a weakened odd-even effect is partly counteracted by a stronger water uptake caused by a stronger relative degradation of the PDADMAC-terminated PEM in comparison to the PSS-terminated PEM.

The question which arises is why is the odd-even effect weakened. The most obvious answer would be that the amount of positive extrinsic binding sites decreases. The excess of positive binding sites is due to an excess of PDADMAC inside PDADMAC-terminated PEMs. Therefore, a probable explanation for the weakened odd-even effect could be simply the release of excess PDADMAC. When the amount of excess PDADMAC is lowered, the odd-even effect should become weaker. However, a higher release of material in comparison to the PSS-terminated sample during the thermal treatment should amplify the decrease of SLD and refractive index, which was not observed. The decrease in SLD and refractive index is the same for all samples. The only way to assume a weakening of the odd-even effect due to exposure of excess PDADMAC is to assume that the excess of PDADMAC is mainly located on the surface of the PEM. If the excess PDADMAC were located on the surface of the PEM, the average SLD and refractive index of the PEM would not be influenced by a release of the PDADMAC.

## 5. Conclusions

The thermally induced structural changes of polyelectrolyte multilayer in dependence of the outermost layer were investigated by neutron reflectivity and ellipsometry. Thereby, the inner and outer parts were observed independently due to partial deuteration of the PEM. The inner block of the PEMs was deuterated while the outermost block was non-deuterated. The thermal treatment causes a partial intermixing of the block structure. Furthermore, the PEMs are affected by two processes simultaneously; a densification and a degradation of the PEM.

The absolute decrease in thickness due to the thermal treatment is similar for all investigated PEMs, which indicates that only a constant outermost part degenerates. It is assumed that the degradation process only influences the diffusion zone of the PEM. The inner part of the PEM densifies due to thermal treatment. The densification mainly affects the restructured zone of the PEM.

In addition, the thermal treatment also influences the swelling behavior of the PEMs. While before the thermal treatment the PDADMAC-terminated PEMs contain much more swelling water than the PSS-terminated PEMs, after the thermal treatment, the differences in swelling water between PSS-terminated and PDADMAC-terminated PEMs decrease slightly for the thin PEMs and vanishes completely for the thick PEMs. Apparently, the odd-even effect becomes weaker due to the thermal treatment. The odd-even effect is caused by excess PDADMAC which can be found in PDADMAC-terminated PEMs. The reason might be that the excess PDADMAC is expelled due to the thermal treatment.

PEMs are interesting candidates for application purposes, like sensors. In many sensor devices, changes in temperature, ionic strength and pH play an important role. All these parameters affect the structure of PEMs. For construction of PEM sensors, one has to be aware that the probed parameter could change the structure and swelling ability irreversibly. The present study shows what kind of structural changes can be expected if a PEM is expelled to high temperatures, which is helpful for the construction of PEM containing devices designed for high operation temperatures.

## Figures and Tables

**Table 1:** Thickness (*d*), refractive index (*n*) and scattering length density (SLD) of the samples measured by ellipsometry and neutron reflectometry. The samples were measured in dried nitrogen (1% RH) and in water (H${}_{2}$O for ellipsometry and D${}_{2}$O for NR) before (b.t.) and after (a.t.) the temperature treatment.

**Conditions**	**20 Layers**			
	$d_{\mathrm{elli}}$ **(nm)**	$n_{\mathrm{elli}}$	$d_{\mathrm{NR}}$ **(nm)**	${\mathrm{SLD}}_{\mathrm{NR}}$ **(10**${}^{-6}$ Å${}^{-2}$**)**
1% b.t.	31 ± 1	1.54 ± 0.01	32.8 ± 0.7	1.55 ± 0.03
water b.t.	55 ± 3	1.468 ± 0.005	56.6 ± 0.9	3.6 ± 0.1
1% a.t.	29 ± 1	1.52 ± 0.01	24.9 ± 0.5	1.13 ± 0.02
water a.t.	53 ± 2	1.462 ± 0.004	54.1 ± 0.8	3.9 ± 0.1
**Conditions**	**21 Layers**			
	$d_{\mathrm{elli}}$ **(nm)**	$n_{\mathrm{elli}}$	$d_{\mathrm{NR}}$ **(nm)**	${\mathrm{SLD}}_{\mathrm{NR}}$ **(10**${}^{-6}$ Å${}^{-2}$**)**
1% b.t.	37 ± 1	1.55 ± 0.01	40.0 ± 0.8	1.71 ± 0.04
water b.t.	60 ± 3	1.488 ± 0.004	59.8 ± 0.9	3.3 ± 0.1
1% a.t.	34 ± 1	1.52 ± 0.01	31.2 ± 0.6	1.12 ± 0.02
water a.t.	59 ± 3	1.472 ± 0.003	60.6 ± 0.9	3.7 ± 0.1
**Conditions**	**36 Layers**			
	$d_{\mathrm{elli}}$ **(nm)**	$n_{\mathrm{elli}}$	$d_{\mathrm{NR}}$ **(nm)**	${\mathrm{SLD}}_{\mathrm{NR}}$ **(10**${}^{-6}$ Å${}^{-2}$**)**
1% b.t.	92 ± 4	1.553 ± 0.003	90 ± 2	1.11 ± 0.02
water b.t.	164 ± 6	1.470 ± 0.002	174 ± 4	4.02 ± 0.07
1% a.t.	89 ± 4	1.544 ± 0.003	79 ± 2	0.97 ± 0.02
water a.t.	142 ± 5	1.486 ± 0.002	136 ± 3	3.47 ± 0.08
**Conditions**	**37 Layers**			
	$d_{\mathrm{elli}}$ **(nm)**	$n_{\mathrm{elli}}$	$d_{\mathrm{NR}}$ **(nm)**	${\mathrm{SLD}}_{\mathrm{NR}}$ **(10**${}^{-6}$ Å${}^{-2}$**)**
1% b.t.	94 ± 3	1.559 ± 0.002	105 ± 3	1.21 ± 0.02
water b.t.	154 ± 4	1.483 ± 0.001	165 ± 5	3.3 ± 0.1
1% a.t.	91 ± 3	1.553 ± 0.001	94 ± 2	1.06 ± 0.02
water a.t.	147 ± 4	1.488 ± 0.001	162 ± 5	3.2 ± 0.1

**Table 2:** The swelling water ($\phi_{\mathrm{swell}}$) and void water ($\phi_{\mathrm{void}}$) before (b.t.) and after (a.t.) thermal treatment, measured from $d_{NR}$ and SLD.

**Conditions**	**20 Layers**		**21 Layers**	
	$\phi_{\mathrm{swell}}$	$\phi_{\mathrm{void}}$	$\phi_{\mathrm{swell}}$	$\phi_{\mathrm{void}}$
D${}_{2}$O b.t.	0.42 ± 0.04	0.02 ± 0.01	0.33 ± 0.04	0.03 ± 0.01
D${}_{2}$O a.t.	0.54 ± 0.05	0.00 ± 0.01	0.49 ± 0.04	0.01 ± 0.01
**Conditions**	**36 Layers**		**37 Layers**	
	$\phi_{\mathrm{swell}}$	$\phi_{\mathrm{void}}$	$\phi_{\mathrm{swell}}$	$\phi_{\mathrm{void}}$
D${}_{2}$O b.t.	0.48 ± 0.04	0.06 ± 0.01	0.38 ± 0.04	0.03 ± 0.01
D${}_{2}$O a.t.	0.42 ± 0.05	0.03 ± 0.01	0.42 ± 0.04	0.00 ± 0.01

## Abbreviations

The following abbreviations are used in this manuscript:

PE: polyelectrolyte
PSS: polystyrene sodium sulfonate
d-PSS: deuterated polystyrene sodium sulfonate
PEI: polyethyleneimine
PDADMAC: poly(diallyldimethylammoniumchloride)
NR: neutron reflectometry
SLD: scattering length density
*n*: refractive index
*d*: thickness
